# Recurrent *Clostridium difficile* infection among Medicare patients in nursing homes: A population-based cohort study

**DOI:** 10.1097/MD.0000000000006231

**Published:** 2017-03-10

**Authors:** Marya D. Zilberberg, Andrew F. Shorr, William M. Jesdale, Jennifer Tjia, Kate Lapane

**Affiliations:** a EviMed Research Group, LLC, Goshen; b School of Public Health and Health Sciences, University of Massachusetts, Amherst, MA; c Washington Hospital Center, Washington, DC; d University of Massachusetts Medical School, Worcester, MA, USA.

**Keywords:** *C. difficile*, costs, hospitalization, Medicare, nursing home, recurrence

## Abstract

We explored the epidemiology and outcomes of *Clostridium difficile* infection (CDI) recurrence among Medicare patients in a nursing home (NH) whose CDI originated in acute care hospitals.

We conducted a retrospective, population-based matched cohort combining Medicare claims with Minimum Data Set 3.0, including all hospitalized patients age ≥65 years transferred to an NH after hospitalization with CDI 1/2011-11/2012. Incident CDI was defined as ICD-9-CM code 008.45 with no others in prior 60 days. CDI recurrence was defined as (within 60 days of last day of CDI treatment): oral metronidazole, oral vancomycin, or fidaxomicin for ≥3 days in part D file; or an ICD-9-CM code for CDI (008.45) during a rehospitalization. Cox proportional hazards and linear models, adjusted for age, gender, race, and comorbidities, examined mortality within 60 days and excess hospital days and costs, in patients with recurrent CDI compared to those without.

Among 14,472 survivors of index CDI hospitalization discharged to an NH, 4775 suffered a recurrence. Demographics and clinical characteristics at baseline were similar, as was the risk of death (24.2% with vs 24.4% without). Median number of hospitalizations was 2 (IQR 1–3) among those with and 0 (IQR 0–1) among those without recurrence. Adjusted excess hospital days per patient were 20.3 (95% CI 19.1–21.4) and Medicare reimbursements $12,043 (95% CI $11,469–$12,617) in the group with a recurrence.

Although recurrent CDI did not increase the risk of death, it was associated with a far higher risk of rehospitalization, excess hospital days, and costs to Medicare.

## Abstract 2

Supplemental Digital Content is available in the text

## 1 Introduction

One of the most challenging aspects in the care of patients with *Clostridium difficile* infection (CDI) is its potential to recur. In some cases recurrence follows exposure to additional courses of antimicrobials, while in others there is no clear inciting event. In general, multiple lines of evidence, including a meta-analysis, randomized controlled trials of CDI therapies, and cohort studies, indicate that the rates of recurrence in this infection range from approximately 10% in hospitalized populations to 50% in other groups.^[1–4]^

Older age represents a well-established risk factor for developing CDI.^[5–9]^ In turn, this suggests that a large and growing proportion of the population faces some risk for a CDI recurrence. Due to age and frailty, the elderly are more prone than the young to come in contact with a nursing home (NH), where the overall prevalence of a CDI diagnosis is 2.1% and rates of asymptomatic carriage of toxigenic *C. difficile* are nearly 50%.^[10,11]^ Despite such high colonization rates, studies suggest that a small minority of CDI in NHs originates from long-term NH dwellers. In fact, up to 90% of all NH-related CDI can be traced to patients transferred to the NH from acute care hospitals.^[11–14]^

Little is known about this population of patients, who, by surviving CDI in acute care, serve as a potential reservoir for spreading this infection within NHs after transfer. Furthermore, to the best of our knowledge no study has examined the risk of recurrence specifically in this group. Understanding the epidemiology and outcomes of recurrence among these patients is crucial in order to improve infection control practices in NHs, and to develop strategies to limit the costs associated with recurrences.

Therefore, we examined the frequency of recurrent CDI and its relationship with hospitalization, mortality, and costs in a group of NH residents who have survived an incident episode of hospitalization with CDI.

## 2 Methods

### 2.1 Study design

We conducted a retrospective, population-based matched cohort study of NH residents hospitalized with CDI and discharged to a nursing facility after the index event. Episodes of CDI were identified using a validated case identification approach (described below). We explored the research identifiable Medicare claims data (enrollment file and parts A, B, and D) linked to Minimum Data Set (MDS) 3.0 data. This study was approved by the University of Massachusetts Medical School Institutional Review Board.

### 2.2 Description of data sources

See Supplemental Appendix 1 for detailed data source description.

### 2.3 Cohort definition

**Figure 1:**
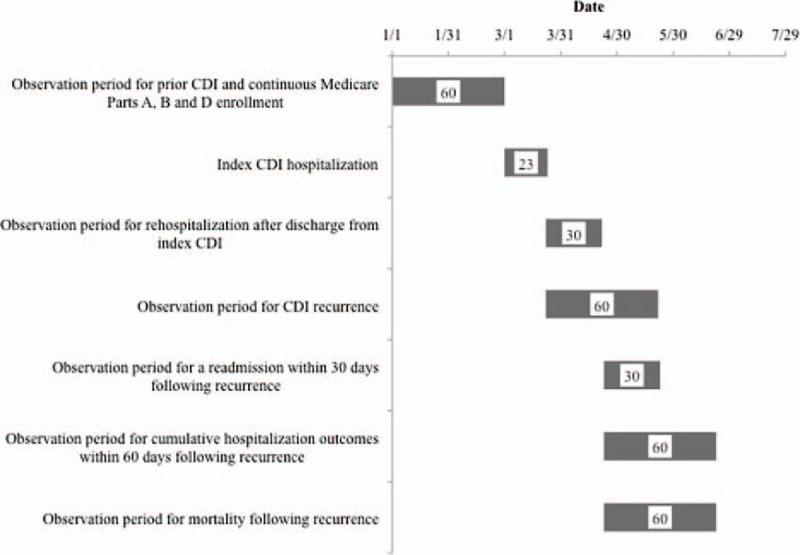
Enrollment and observation timeline for a hypothetical patient in the cohort^∗^. CDI = *Clostridium difficile* infection. Pathway for a hypothetical patient enrolled in the cohort. This patient's index hospitalization with an incident CDI begins on March 1 after 60 days of observation to establish appropriate Medicare enrollment and CDI-free period. Discharge occurs on hospital day 23, when the observation period for 30-day rehospitalization as well as a recurrence within 60 days commences. In this hypothetical patient, a recurrent CDI is noted on day 30 following index discharge. At this point, observation period for a 30-day rehospitalization and 60-day mortality begins. ^∗^Each block signifies duration of the corresponding event (numbers inside are days).

The cohort consisted of elderly (age ≥65 years) patients from the entire population of the United States and its Atlantic island territories. Subjects had to survive a hospitalization with an incident CDI episode in an acute care setting and to be discharged to an NH (Fig. 1). We further required a full NH admission MDS assessment. All index hospitalizations occurred between March 1 and December 31, 2011, after 60 days of both continuous co-enrollment in Medicare parts A, B, and D and absence of evidence of CDI on MDS or inpatient records. An index episode of CDI was defined by the presence of an ICD-9-CM code of 008.45 on any hospitalization at an acute care facility during the March to December period.

### 2.4 CDI episodes

CDI was considered incident if there was no evidence of CDI in the previous 60 days. Each incident CDI episode was classified as community-acquired (CA) if there was a CDI code in the “present on admission” field, and no evidence of a previous admission to another hospital, skilled nursing facility (SNF), or an NH within 12 weeks of the index admission date.^[15]^ A community-onset healthcare facility associated event occurred if there was a CDI code in the “present on admission” field, and the patient was transferred from another healthcare facility or had evidence of admission to another hospital, SNF, or an NH within 4 weeks of the index admission date. The index CDI episode was deemed hospital-onset healthcare facility associated if there was no CDI code in the “present on admission” field.^[16]^ Those patients with a CDI code in the “present on admission” field and evidence of admission to another hospital, SNF, or a NH within 4 to 12 weeks of the index admission date were classified as “indeterminate.”

CDI recurrence was defined by one of the following occurring within 60 days from index discharge or of the last day of incident CDI treatment in the NH: 1. Note of treatment with oral metronidazole, oral vancomycin, or fidaxomicin for at least 3 days at the NH in the part D file, or 2. An ICD-9-CM code for CDI (008.45) during a repeat hospitalization. To distinguish a recurrence from persistence, we required a gap of at least 7 days in drug administration at the NH after discontinuation of treatment for the index episode of CDI.

### 2.5 Main exposure and covariate measures

Demographic (age, gender, and race/ethnicity) and clinical (acute illness severity [sepsis, toxic megacolon, bowel perforation, colectomy, and intensive care unit stay] and comorbidity burden [individual comorbidities and Charlson comorbidity index]) characteristics for the cohort were collected during the index hospitalization.

### 2.6 Outcome measures

We examined rehospitalization rates within 30 days of a recurrence, time to rehospitalization, hospital length of stay (LOS) and costs (measured as Medicare payments in $US) for the initial postrecurrence rehospitalization, and 60-day mortality in patients with recurrent CDI compared to those without (Fig. 1). The observation period was extended to 60 days to compute the number of hospitalizations per patient over time, and cumulative per-patient hospital days and costs. The latter 2 outcome variables were derived by summing across all part A events (hospitalizations at acute care facilities, other types of hospitals, and Medicare-reimbursed short-stay SNF stays) that began within 60 days of the onset of recurrence, including those hospitalizations that defined the recurrence event, and those that extended beyond the end of the 60 day window.

### 2.7 Statistical analyses

We used descriptive statistics to characterize the group of NH residents with a recurrent CDI who were matched (with replacement) to those without a recurrence based on the date of discharge from the index hospitalization in order to create comparable time periods of observation. Patients were matched on discharge date from the index hospitalization, conditional on survival until the matched case's recurrence date, to ensure comparable levels of follow-up available for outcome ascertainment from the recurrence date forward. We used the GREEDY algorithm to match as many controls as possible to each case with replacement.^[17]^ Thus, the 9704 potential controls could be matched to more than 1 case, resulting in 13,736 matches, with a range of 1 to 25, and a median of 3 matches per case. Those without a recurrence were eligible for inclusion if they were alive on the day recurrence was noted in the comparator group, either via a repeat CDI ICD-9-CM code or based on medication usage at the NH. Due to the limited utility of statistical testing in a sample this large, we elected to consider a prevalence difference of 5% worthy of note. Although the clinical difference of 5% is somewhat arbitrary, it is well within the range that most clinicians would consider an important difference.

We derived Cox proportional hazards (to examine 30-day rehospitalization and 60-day mortality risks) and linear (to compute excess cumulative 60-day hospital days and costs) models adjusting for age, gender, race, and comorbidities, in patients with recurrent CDI compared to those without a recurrence.

## 3 Results

### 3.1 Recurrence rates and baseline patient characteristics

Among 14,472 patients who survived their index CDI hospitalization and were discharged to a NH, 4775 (33.0%) suffered a recurrence. Of those, 4768 (99.9%) were matched to 13,736 without a recurrence (weighted to 4768). Those with and without a recurrence were similar in age, gender, and race/ethnicity (Table 1). Similarly, there were no substantive differences between the groups in the distribution of comorbid conditions or the Charlson comorbidity index. Although those without a recurrence were more likely to have their index CDI classified as hospital-acquired than those with a subsequent recurrence (25.2% vs 18.3%), the situation was reversed for the unknown acquisition category (27.7% vs 32.6%, respectively) (Table 1).

**Table 1:**
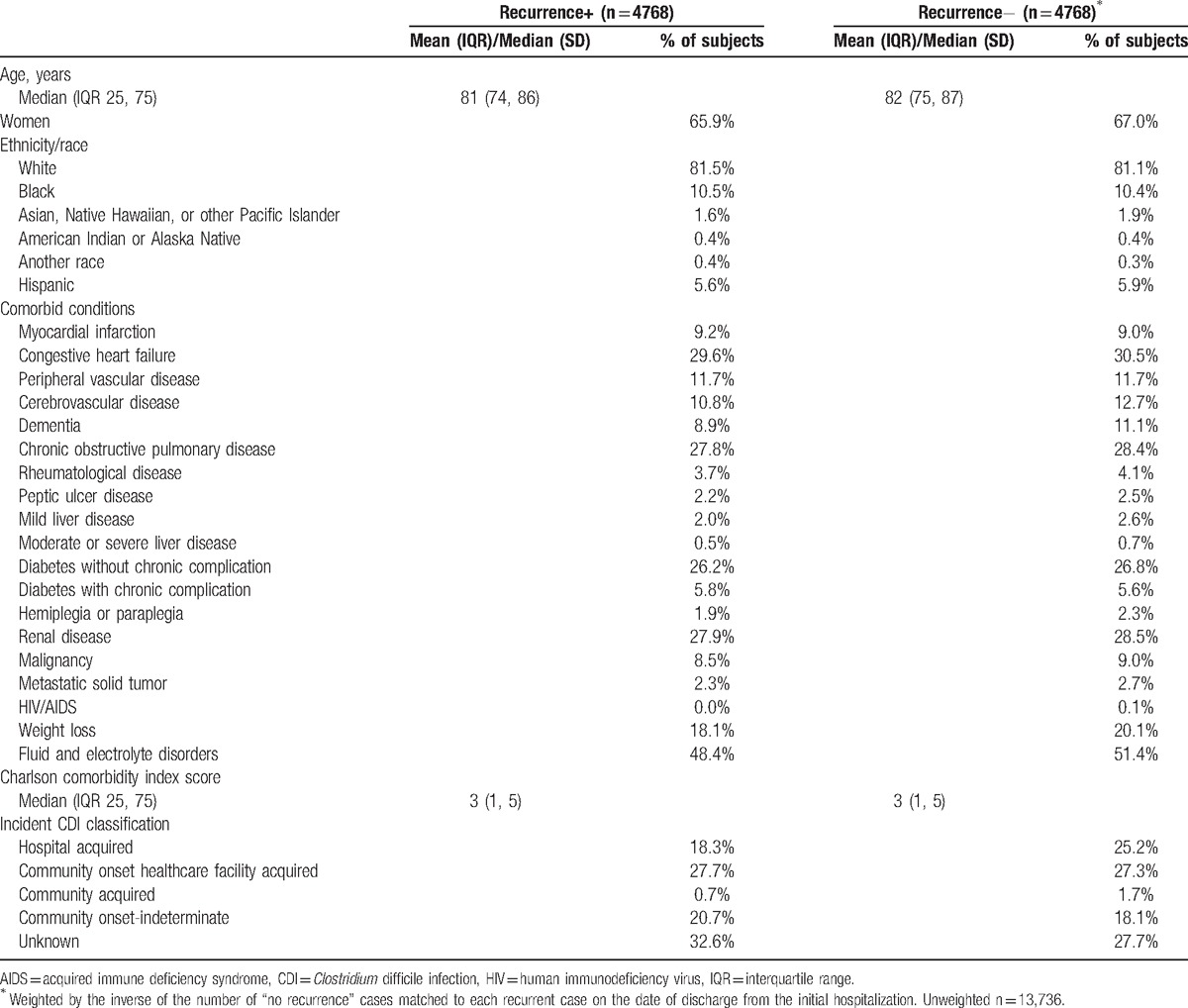
Characteristics at the time of index hospitalization of patients with and without a recurrent episode of *Clostridium difficile* infection.

### 3.2 Events during index hospitalization

Although frequency of sepsis during the index hospitalization was similar in the 2 groups, the need for intensive care unit care was more common among those who did not (29.0%) than those who did (24.5%) experience a recurrence (Table 2). Although the rate of colectomy was 2-fold higher in the nonrecurrent than recurrent group, this procedure was rare in both (Table 2). As for unadjusted hospital LOS and costs of the index hospitalization, both were numerically higher in the non-recurrence group than in those with a recurrence (Table 2). Notably, however, the risk of rehospitalization within 30 days of the index discharge among patients with any eventual CDI recurrence during our study time horizon (64.9%) was more than triple that among those without (17.8%).

**Table 2:**
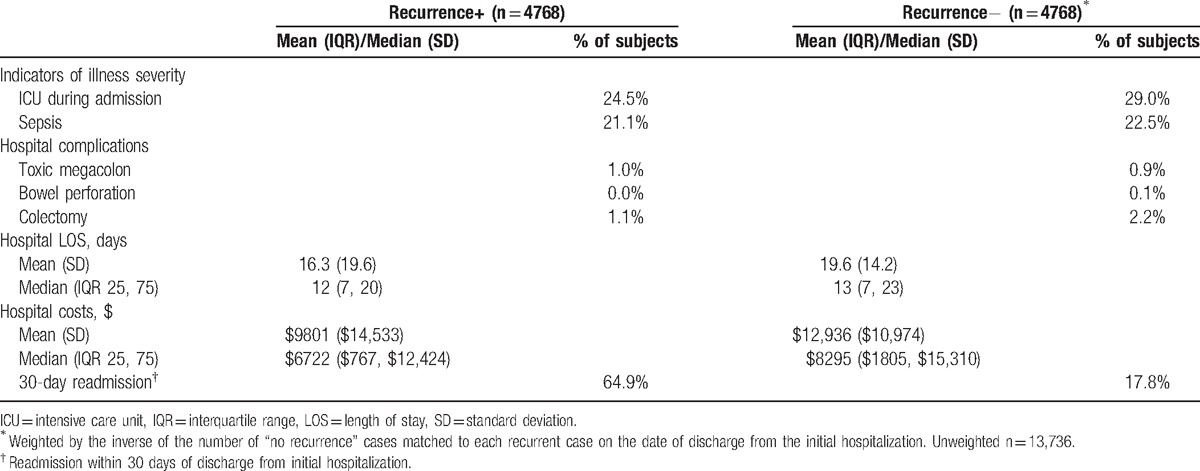
Index hospitalization illness severity, complications, and outcomes.

### 3.3 Recurrence outcomes

In contrast, within 30 days of the CDI recurrence (Fig. 1), hospitalization occurred in 28.1% of the individuals compared to 17.8% in the same timeframe among those without a recurrence, and median time to hospitalization was similar between the 2 groups (13 days with recurrence vs 12 without) (Table 3). The 60-day mortality in the 2 groups was also similar (24.2% vs 24.4%).

**Table 3:**
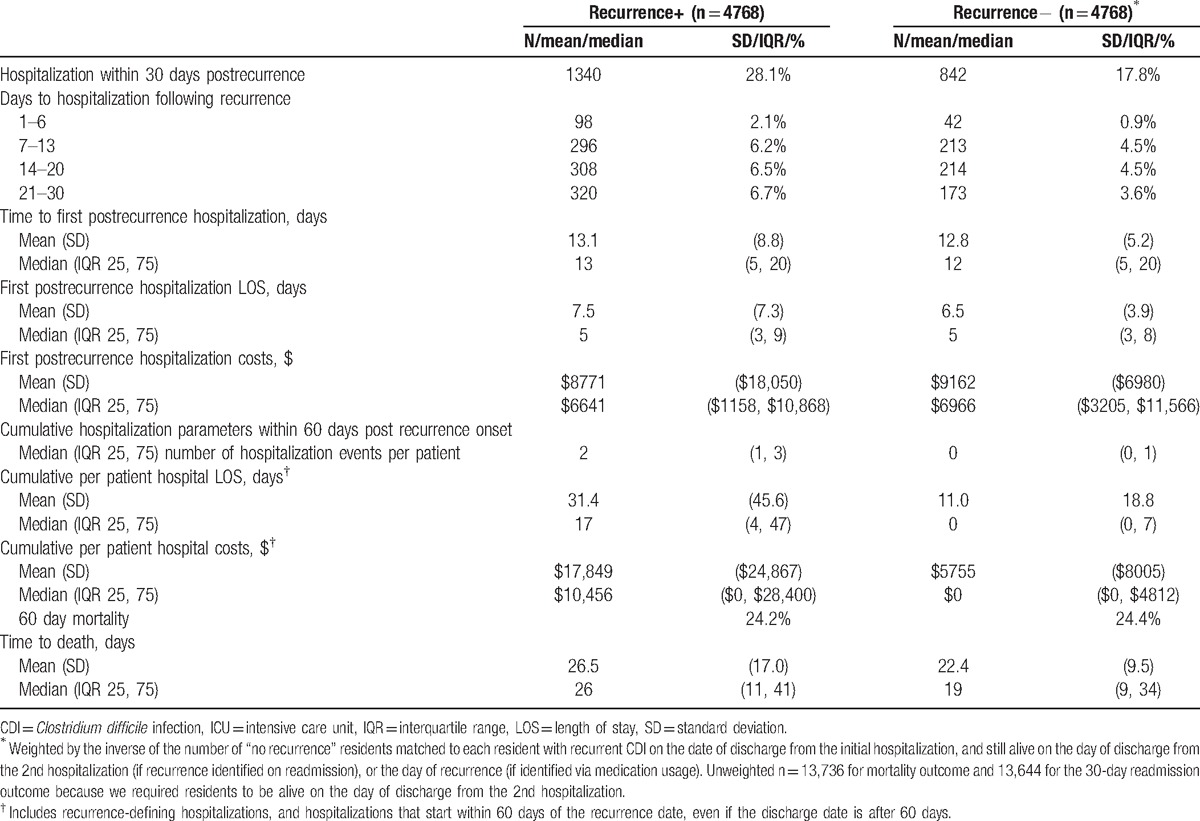
Unadjusted outcomes among those with and without CDI recurrence.

In the adjusted analysis, CDI recurrence was associated with a 67% (95% CI 55%–79%) increase in the hazard of 30-day rehospitalization (Table 4). Although neither hospital LOS nor costs per first postrecurrence hospitalization differed substantively among those who were rehospitalized, the cumulative burden of hospitalizations in the group that recurred far exceeded that in the group that did not (Table 3). Consistent with this, the adjusted excess hospital days per patient were 20.3 (95% CI 19.1–21.4) and costs were $12,043 (95% CI $11,469–$12,617) compared to those without a recurrence (Table 4).

**Table 4:**
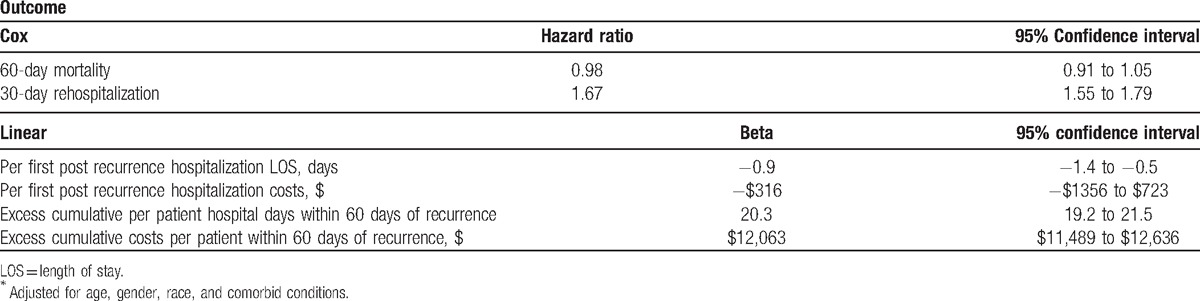
Recurrent CDI-attributable outcomes^∗^.

## 4 Discussion

In the current study of older patients whose incident CDI occurred during a hospitalization that ended in a discharge to an NH, all survivors of such hospitalization had a 33% risk of developing a recurrent bout of CDI within 60 days following discharge or termination of anti-CDI therapy. Although there were no dramatic differences noted during the index hospitalization between those who recurred and those who did not, the group with an eventual recurrence had a nearly 4-times the risk of those without a recurrence of a 30-day readmission following discharge from the index hospitalization, whether with the CDI recurrence or preceding it. Similarly, the risk of a repeat hospitalization following recurrent CDI was significantly higher in the group with than the matched group without a recurrence. Although per hospitalization LOS and costs did not differ between the groups, because of the increased likelihood of admission in the recurrence group, the cumulative 60-day excess rehospitalization days (∼20) and costs (∼$12,000) were high, suggesting the economic burden for Centers for Medicare and Medicaid Services in conjunction with CDI recurrence in the NH population is substantial and likely to expand with the aging of the population.

Pepin et al^[18]^ examined the course of recurrent CDI in a Canadian cohort of over 460 patients treated between 1991 and 2005. Of these, 9.3% suffered death within 30 days of recurrence. A more recent mixed (in- and outpatient) cohort study from Scotland found 16.4% mortality within 1 year of the onset of incident infection in those with a recurrence compared to only 0.5% among those without.^[19]^ Last, Olsen et al^[20]^ examined 180-day mortality in a cohort of patients with a CDI that recurred following an incident CDI hospitalization. In this investigation, a recurrence raised the adjusted risk of death by over 33%. In contrast, we did not detect a difference in 60-day mortality. This important divergence suggests that, at the very least, age (our cohort is substantially older than others) and possibly non-CDI illness burden (marked by needing a discharge to a NH following their index hospitalization), may modify the effect of CDI recurrence on mortality. Although it would be of interest to examine causes of death, we did not have access to this information.

Only a handful of studies have taken a detailed look at rehospitalizations associated with CDI recurrence among patients with CDI.^[21–23]^ In one from a large urban academic medical center, among nearly 4000 patients with an incident CDI, 11% developed a documented recurrence.^[21]^ Patients with a recurrence (85%) were approximately twice as likely as those without (41%) to require a rehospitalization within 180 days following their initial bout of CDI. The 2nd study focused on critically ill patients by asking whether an early recurrence of CDI impacts 30-day rehospitalization.^[22]^ Indeed the investigators reported a significant elevation in the risk of rehospitalization in the face of a recurrence, with the adjusted odds ratio exceeding 15. Our numbers, though more modest, comport with those studies in terms of the relative rise in readmission associated with CDI recurrence. The far lower magnitude of the problem as detected in the current study is likely due to a combination of several factors. First, there were important population differences, particularly given that one of the studies was confined to the critically ill. Second, our observation timeframe of 60 days was only 1/3 of Olsen et al. It is likely that with a longer observation period we would have detected a higher rate of readmission in both groups.

Olsen et al^[21]^ additionally compared numbers of hospitalizations and numbers of hospital days in the 180-day follow-up period between the group that experienced a recurrence and one that did not. In each comparison, the group with a recurrence fared worse than the group without. Namely, the group with a recurrence incurred an excess of 11 days over those without, and the number of readmissions per patient was 1.72 versus 0.81 for the groups, respectively. Recurrent CDI had an independent impact on these outcomes. Our observations are consistent with those investigators’ in that the group with a recurrence was substantially more likely to experience a rehospitalization, and this resulted in an average excess of 20 days in the hospital for a patient with a recurrence over 60 days following the onset of said recurrence. Although this number of excess days is higher than previously reported, it is not surprizing given the elderly NH population in our study.

Finally, Dubberke et al^[23]^ in a single center cohort study of general hospital population reported the costs attributable to recurrent CDI over 180 days to be ∼$11,000. Given the age and illness severity of our population, it is not surprizing that our estimate was similarly high over a much shorter period of time. Additionally, in contrast to Dubberke, our >$12,000 excess in hospital costs (more accurately, Medicare reimbursement) per patient in the presence of a recurrence includes not only acute, but also other short-term hospitalizations.

Our study has a number of limitations. As a retrospective study it is subject to a number of biases, most notably selection bias. To mitigate this we developed a priori inclusion criteria. Because we used administrative coding to identify incident CDI, there is a threat of misclassification, though this method of identifying CDI is well validated in the hospitalized population.^[24]^ One clue to the extent that it is present is the fact that toxic megacolon, a complication specific to CDI, was not confined to the CDI group, and occurred, albeit with a lower frequency, among non-CDI patients. However, such misclassification likely biased our results toward the null. Using the ICD-9-CM code for detection of recurrent CDI is potentially thornier, as this code may or may not signify the actual presence of active CDI. Wen et al,^[25]^ in a validations study exploring ways to identify CDI recurrence in administrative datasets, reported a relatively low specificity and, consequently, positive predictive value of a combination of ICD-9 codes, stool-testing procedure codes, and CDI treatment codes for recurrent CDI. This raises the possibility that a substantial proportion of those identified as CDI recurrence were, in fact, related to the history of CDI rather than an active infection. The extent of this misclassification is difficult to estimate, as the nature of our study design (all US hospitals as opposed to two academic urban centers) and population (elderly patients discharged to an NH after a CDI hospitalization versus all ages followed in the outpatient clinics affiliated with the academic centers) may invalidate comparisons. Using the MDS dataset to identify CDI presented its own challenges. Because the current version of MDS does not explicitly list CDI as an entity, we had to develop an algorithm to identify CDI recurrence. Although this algorithm has never been validated, by requiring CDI-specific treatment or a rehospitalization with the principal diagnosis of CDI we attempted to increase the specificity of case identification. However, misclassification may have led to underestimation of the rate of recurrence. A source of additional potential misclassification relates to our inability to identify either the dose or the duration of treatment of the incident CDI episode, and, thus, inadequately treated cases may have contributed to recurrence. However, this mimics closely the potential impact of under-treatment on recurrence in real-world clinical practice.

An additional point where misclassification may have occurred is the 60-day postindex discharge period. Because, unless present on admission, it is unknown when incident CDI occurred during the index hospitalization, it is possible that, particularly when the index hospitalization itself was lengthy, what we classify as a recurrence is in fact a de novo case of CDI. The fact that our recurrence rate was well within the range reported in the literature is reassuring.^[26]^ Additionally reassuring is the fact that 33% is on the higher end of this range, as would be expected in an older and sicker population. Similarly, this definition of a recurrence may account for the high rate of rehospitalization in the recurrence group. At the same time, it is possible that the null mortality result is explained at least in part by this high risk of misclassification.

Confounding is an issue in observational studies. Although we dealt with the possibility of confounding by deriving regression models to estimate the effect of CDI on the outcomes of interest, the possibility of residual confounding remains. Because the timing of events during the index hospitalization was not available, we had to infer that such events as bowel perforation and colectomy represented complications of incident CDI. It is, however, possible that they preceded its occurrence.

These limitations notwithstanding, the size and generalizability of the dataset, as well as our rigorous statistical methods shed needed light on CDI recurrence in this important population. However, our definitions, and specifically that of recurrent CDI, require further validation in a clinical dataset that resembles population examined here.

At the same time, this study has some major strengths. As the largest study of its kind, drawing from the entire Medicare population of the US, its results are highly generalizable. The novelty of the population examined is of interest. Although these are the patients known to bring CDI into NHs, ours is the first undertaking to address this population comprehensively. Finally, by reporting the actual Medicare reimbursements associated with treatment of this disease, we underscore the financial pressures, in addition to clinical ones, on institutions to target aggressive CDI prevention measures in this population.

In summary, in the largest study of the Medicare population addressing a group of patients that acts as a potential reservoir of CDI in NHs, we have detected a high rate of recurrent CDI. Although we did not find differences in either 60-day mortality, hospital LOS, or costs per individual rehospitalization event, recurrent CDI had an important association with the risk of rehospitalization, thus adding to the cumulative strain on healthcare resources. Strategies shown to reduce CDI recurrence should be evaluated as a way not only to improve patient outcomes, but also to ease the burden on the healthcare system.

## Supplementary Material

Supplemental Digital Content
