# Supplementary material for: Recurrent Clostridium difficile infection among Medicare patients in nursing homes: A population-based cohort study
Source: Medicine (Baltimore). 2017 Mar 10;96(10):e6231. doi: 10.1097/MD.0000000000006231 (PMC5348165; doi:10.1097/MD.0000000000006231)
Supplement: Supplemental Digital Content [file medi-96-e6231-s001.docx]

**Appendix 1. Data source**

The Minimum Data Set (MDS), version 3.0 (2011-2012)

The MDS is completed for each Medicare patient on NH admission, quarterly and annually, and upon major changes in status. MDS assessment is a federally mandated, validated instrument that assesses health conditions, disease diagnoses, treatments, and functional and cognitive status of residents living in all licensed US NHs.

Medicare Provider and Analysis Review (MedPAR) (2011-2012)

The MedPAR files contain final action fee-for-service claims data submitted by inpatient hospital and skilled nursing facility providers for reimbursement of facility costs. By linking this file to MDS and other Medicare files, we were able to identify each episode of CDI and follow the patient along the spectrum of care over time.

Medicare Part D file (2011)

The Medicare Part D event file contains prescription transactions for Medicare beneficiaries. We used it to identify use of medications that increase the risk of CDI (e.g. proton pump inhibitors) and medications to treat CDI (e.g. oral vancomycin), thus providing evidence for the treatment of an initial or refractory episode of CDI.

Medicare Master Beneficiary Summary File (2011-2012)

The Master Beneficiary Summary File includes beneficiary enrollment data, National Death Index cause of death, summarized information about the service utilization and Medicare payment amounts by file type.
